# Supplementary material for: Cohesin mutations are synthetic lethal with stimulation of WNT signaling
Source: eLife. 2020 Dec 7;9:e61405. doi: 10.7554/eLife.61405 (PMC7746233; doi:10.7554/eLife.61405)
Supplement: Figure 5—source data 1. [file elife-61405-fig5-data1.pdf]

Figure 5 - Source Data 1

Chameleon Duo Stain
Protein ladder

pbeta-catenin (Ser33/37/Thr41) - 92 kDa

200 kDa
160 kDa
125 kDa
90 kDa
70 kDa

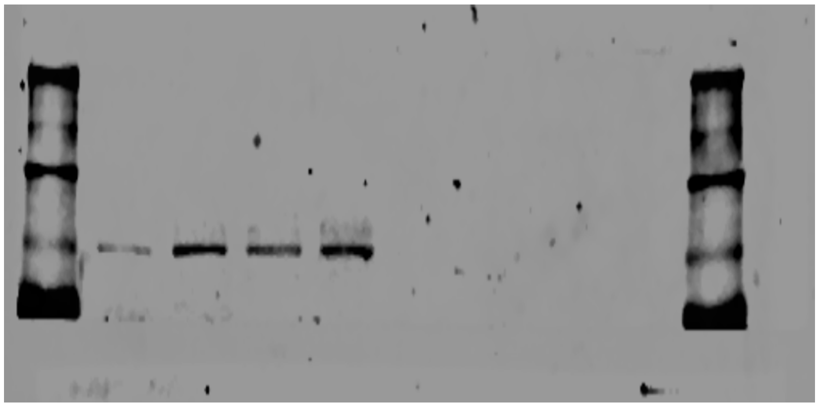

Figure 5A (top)

Total beta-catenin - 92 kDa

90 kDa
(reprobe)

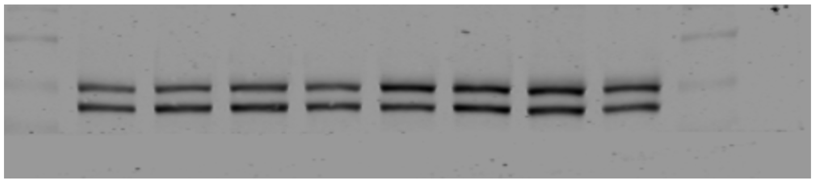

Figure 5A (middle)

Gamma-tubulin - 52 kDa

50 kDa

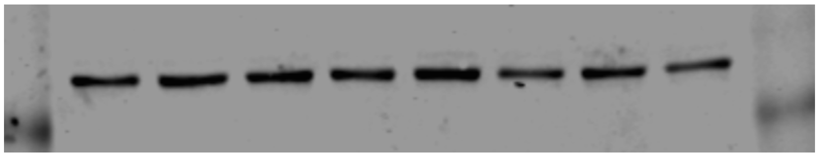

Figure 5A (bottom)

Chameleon Duo Stain
Protein ladder

pbeta-catenin (Ser675) - 92 kDa

200 kDa
160 kDa
125 kDa
90 kDa
70 kDa

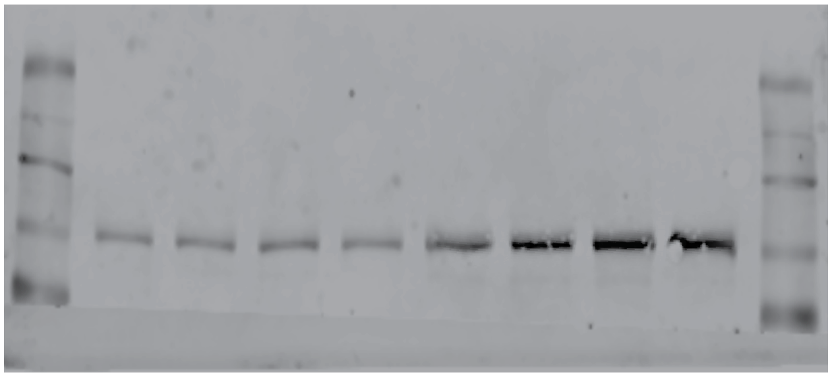

Figure 5B (top)

Total beta-catenin - 92 kDa

90 kDa
(reprobe)

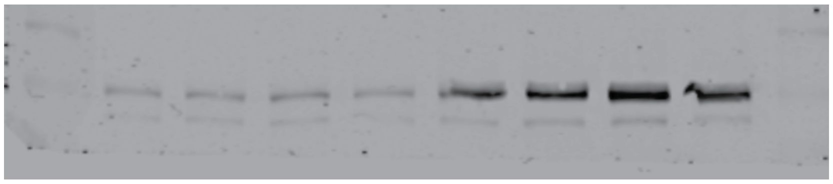

Figure 5B (middle)

Gamma-tubulin - 52 kDa

50 kDa

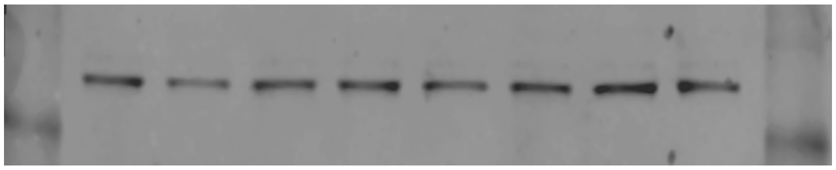

Figure 5B (bottom)
