# Supplementary figures and images for: Cohesin mutations are synthetic lethal with stimulation of WNT signaling
Source: eLife. 2020 Dec 7;9:e61405. doi: 10.7554/eLife.61405 (PMC7746233; doi:10.7554/eLife.61405)

Figure 5 - Source Data 3

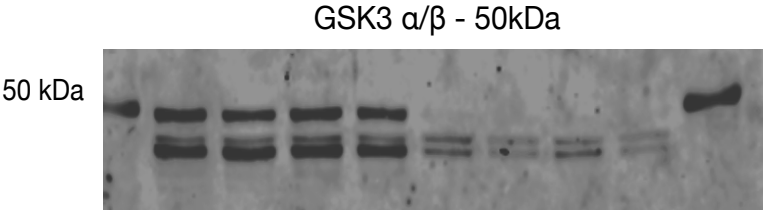

Figure 5S1A (top)

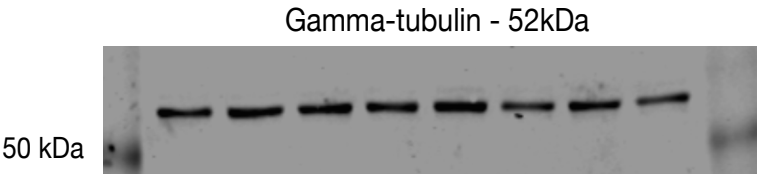

Figure 5S1A (bottom)

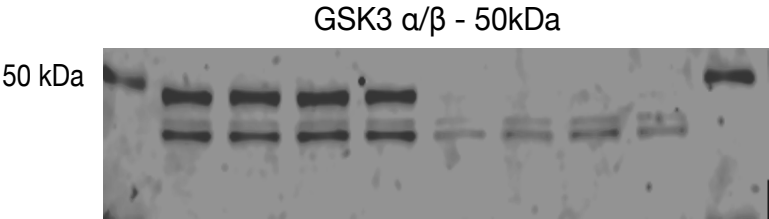

Figure 5S1B (top)

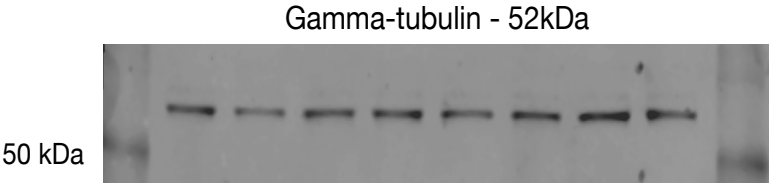

Figure 5S1B (bottom)

Supplement: Figure 5—source data 3. [file elife-61405-fig5-data3.pdf]
