# Supplementary material for: Cohesin mutations are synthetic lethal with stimulation of WNT signaling
Source: eLife. 2020 Dec 7;9:e61405. doi: 10.7554/eLife.61405 (PMC7746233; doi:10.7554/eLife.61405)
Supplement: Figure 5—source data 4. [file elife-61405-fig5-data4.pdf]

Figure 5 - Source Data 4

pbeta-catenin (Ser33/37/Thr41) - 92 kDa

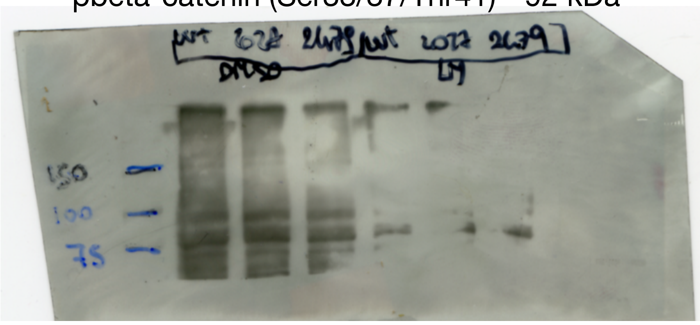

Figure 5S2A (top)

Total beta-catenin - 92 kDa

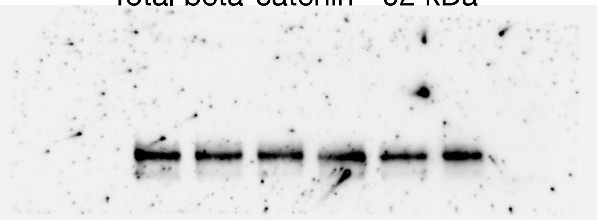

Figure 5S2A (middle)

Gamma-tubulin - 52 kDa

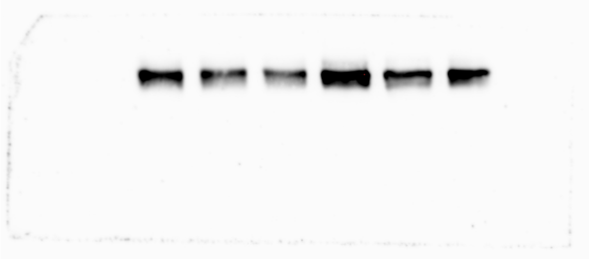

Figure 5S2A (bottom)

pbeta-catenin (Ser675) - 92 kDa

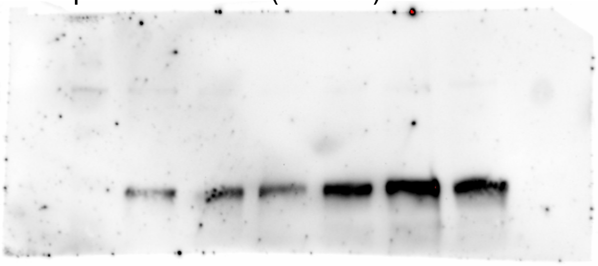

Figure 5S2B (top)

Total beta-catenin - 92 kDa

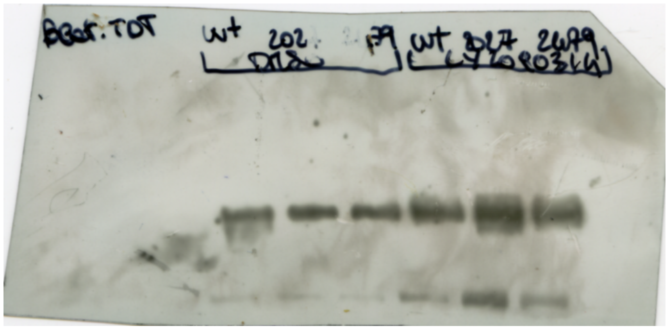

Figure 5S2B (middle)

Gamma-tubulin - 52 kDa

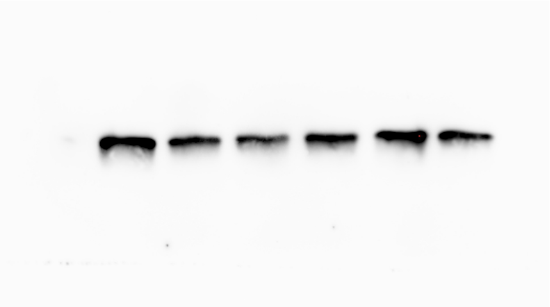

Figure 5S2B (bottom)
