# Supplementary material for: Cohesin mutations are synthetic lethal with stimulation of WNT signaling
Source: eLife. 2020 Dec 7;9:e61405. doi: 10.7554/eLife.61405 (PMC7746233; doi:10.7554/eLife.61405)
Supplement: Supplementary file 1. [file elife-61405-supp1.docx]

| Target gene | Position | sgRNA sequence (5’-3’) | On-target activity (%) |
| --- | --- | --- | --- |
| *RAD21* | 5’UTR | CATCCTGCCGATTTGAACCG**AGG** | 92 |
| *RAD21* | 3’UTR | TACAAACGGGGAAAACTCCT**TGG** | 82 |
| *SMC3* | 5’UTR | TGTACATGATTCCGGGCCCC**TGG** | 94 |
| *SMC3* | 3’UTR | GTAATATGATTCTCATACCC**AGG** | 85 |
| *STAG2* | 5’UTR | TTTCCCCAATGGATCTATGA**AGG** | 87 |
| *STAG2* | 3’UTR | TCCCGCGGTTCTAGAGCGGT**GGG** | 96 |

**Supplementary File 1. List of sgRNA sequences and PCR primers.** This file contains details of the sequence-based reagents in the Key Resources table.

**Supplementary File 1 – Table 1.** sgRNA sequences targeting each of the cohesin subunit genes. The PAM sequences are highlighted in red.

**Supplementary File 1 – Table 2.** Primer sequences used in PCR assay to screen for cells with heterozygous deletion of RAD21 or SMC3 and homozygous deletion of STAG2.

| Target gene | Target sites | Primer | Primer sequence | Expected product (bp) |
| --- | --- | --- | --- | --- |
| *RAD21* | 5’UTR | Forward | ACAGCAGAGATGGCCAAAGAC |  |
|  |  | Reverse | TCTCACTGGAAGAGACTATGACAA | 432 |
|  | 3’UTR | Forward | TTTTGTTTGGCTGAGGGGAGC |  |
|  |  | Reverse | CACACCTCTCGGAGATCCTG | 401 |
|  | 5’UTR/3’UTR | Forward | TTTTGTTTGGCTGAGGGGAGC |  |
|  |  | Reverse | TCTCACTGGAAGAGACTATGACAA | 680 |
| *SMC3* | 5’UTR | Forward | TGGCTAACACCGCAACTCTC |  |
|  |  | Reverse | CCTTGCGAGGTGTAGCTTCC | 327 |
|  | 3’UTR | Forward | GCCCTGCCTACTACAAGGAC |  |
|  |  | Reverse | GGGACCACCACCAACTGAAT | 650 |
|  | 5’UTR/3’UTR | Forward | TGGCTAACACCGCAACTCTC |  |
|  |  | Reverse | GGGACCACCACCAACTGAAT | 450 |
| *STAG2* | 5’UTR | Forward | GGTGCCCCTCCAAACTTCT |  |
|  |  | Reverse | CCCAAACAATCCCGAAGC | 465 |
|  | 3’UTR | Forward | CTTTGCAAGCAGTCTTCAGGT |  |
|  |  | Reverse | TTTTTGGCTACCACAGCCCA | 423 |
|  | 5’UTR/3’UTR | Forward | GGTGCCCCTCCAAACTTCT |  |
|  |  | Reverse | TTTTTGGCTACCACAGCCCA | 850 |

**Supplementary File 1 – Table 3.** Primer sequences used in RT-qPCR.

| Target gene | Primer (Forward) | Primer (Reverse) |
| --- | --- | --- |
| *Cyclophilin* | ACGGCGAGCCCTTGG | TTTCTGCTGTCTTTGGGACCT |
| *GAPDH* | TGCACCACCAACTGCTTAGC | GGCATGGACTGTGGTCATGAG |
| *RAD21* | CAATGCCAACCATGACTCAT | CGGTGTAAGACAGCGTGTAAA |
| *SMC3* | GTTTCAACCCAGCTGGCCCGTG | CGATGGCTGACTTGGTCACATTCCA |
| *STAG2* | ACGGAAAGTGGTTGAGGG | GTGGAGGTGAGTTGTGGTGT |
