# Supplementary material for: Cohesin mutations are synthetic lethal with stimulation of WNT signaling
Source: eLife. 2020 Dec 7;9:e61405. doi: 10.7554/eLife.61405 (PMC7746233; doi:10.7554/eLife.61405)
Supplement: Supplementary file 2. [file elife-61405-supp2.docx]

**Supplementary File 2: TCGA analysis of STAG2 mutant vs wild type cancers**

1. **GSEA gene expression enrichment list**

**
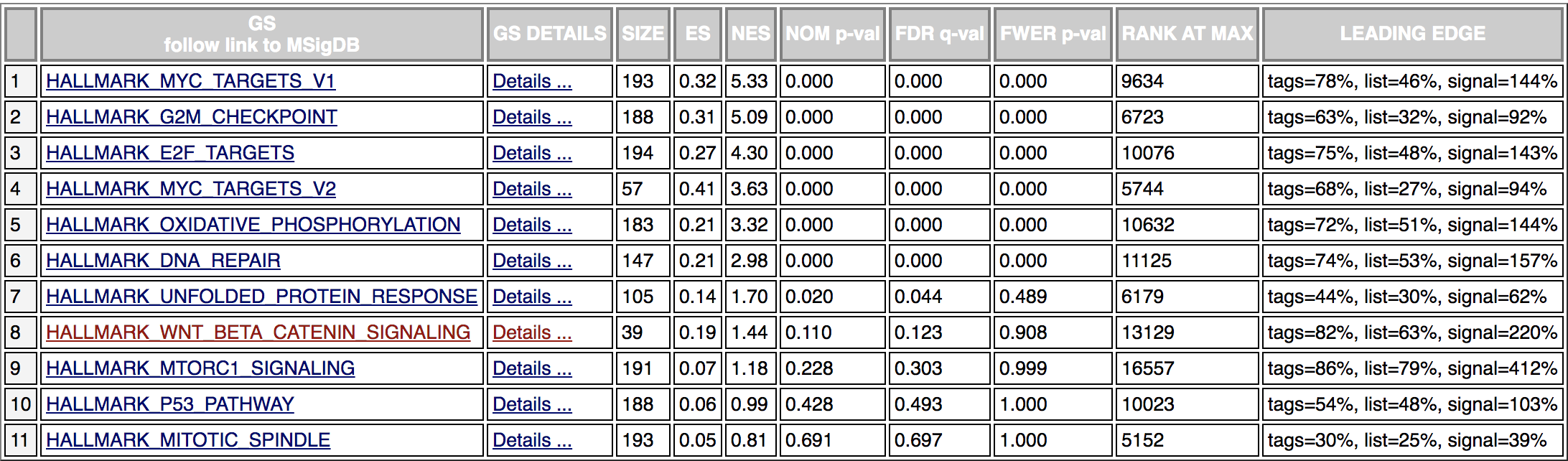
**List of the positively enriched pathways across Bladder Urothelial Carcinoma (BLCA), Uterine Corpus Endometrial Carcinoma (UCEC), Glioblastoma multiforme (GBM) and Cervical Kidney renal papillary cell carcinoma (KIRP) cancers in TCGA that are STAG2 nonsense mutated vs STAG2 intact.

1. **Genes in Hallmark WNT gene list**

<https://www.gsea-msigdb.org/gsea/msigdb/cards/HALLMARK_WNT_BETA_CATENIN_SIGNALING>

| **Ligands** | **Receptors** | **Intermediates** | **Targets** | **Other pathways** |
| --- | --- | --- | --- | --- |
| WNT1, WNT5B, WNT6 | FZD1, FZD8 | AXIN1, AXIN2, CTNNB1, CUL1, DKK1, DKK4, DVL2, GNAI1, LEF1, SKP2, TCF7 | ADAM17, AXIN1, AXIN2, CCND2, CSNK1E, DKK1, DKK4, FRAT1, HDAC11, HDAC2, HDAC5, KAT2A, LEF1, MAML1, MYC, NCOR2, NCSTN, PPARD, TCF7, TP53 | DLL1, HEY1, HEY2, JAG1, JAG2, NOTCH1, NOTCH4, NUMB, PSEN2, PTCH1, RBPJ |

1.
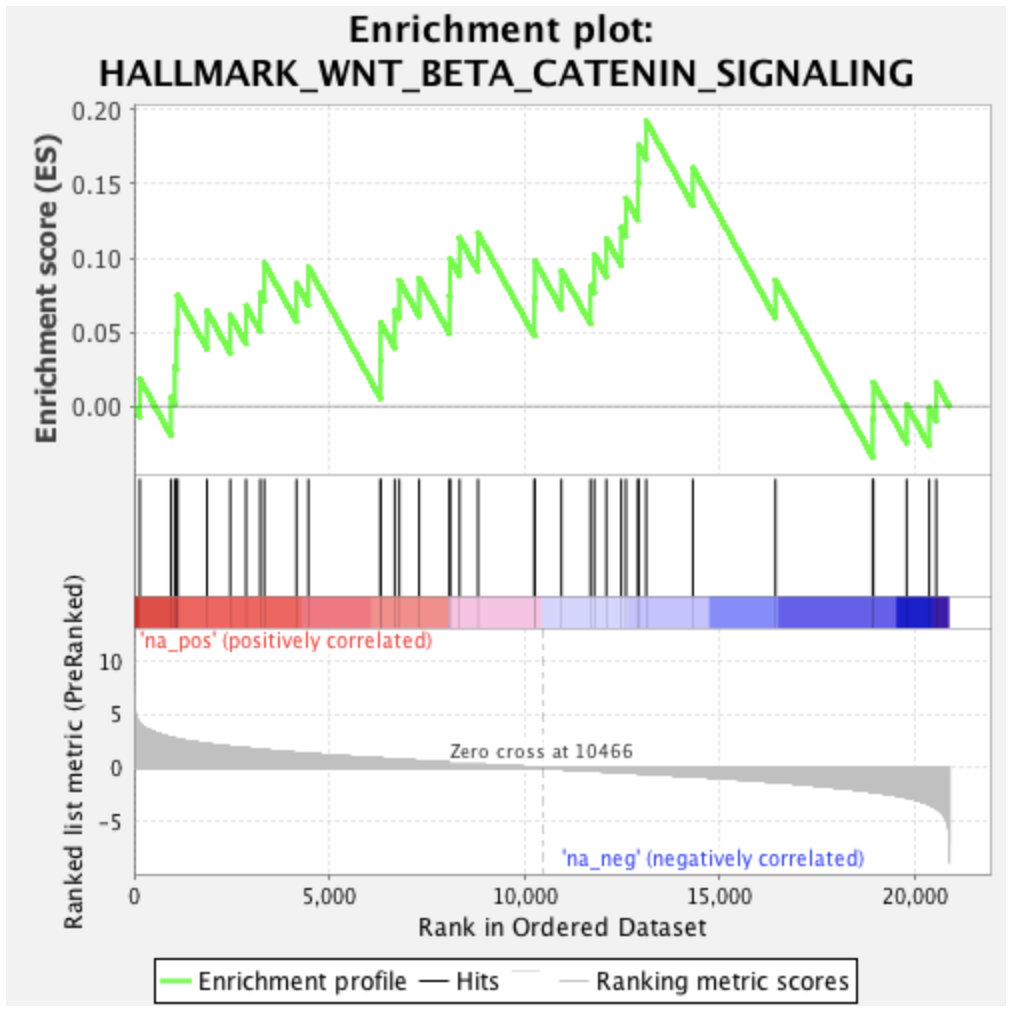
 **Hallmark WNT enrichment plot**

**Method**

*STAG2* nonsense mutations (frame-shift or stop-gained events) with gene expression were retrieved from TCGA [1] by cancer type. Only cancer types having more than two *STAG2* nonsense mutation samples were considered. After this filtering, four cancer types are remaining: Bladder Urothelial Carcinoma (BLCA), Uterine Corpus Endometrial Carcinoma (UCEC), Glioblastoma multiforme (GBM) and Cervical Kidney renal papillary cell carcinoma (KIRP).

For each cancer type, an equal number of control samples were also retrieved. Samples were considered as control if no *STAG2* nonsense mutation was present. Gene Set Enrichment Analysis (GSEA) [2] was performed using Wald statistic ranked approach against MSigDB [3] hallmark gene sets. After ranking the gene expression of the 150 samples from all the cancer type by Wald statistic, *STAG2* was the top ranked gene showing then a strong down-regulation between STAG2 nonsense mutated and controls.

The comparison of this ranking to the total HALLMARK gene sets reveal a positive enrichment (NES: 1.43) with the HALLMARK_WNT_BETA_CATENIN pathway at 8^th^ position of all the gene sets.

**References**

1. Grossman RL, Heath AP, Ferretti V et al. Toward a Shared Vision for Cancer Genomic Data, N Engl J Med 2016;375:1109-1112.

2. Subramanian A, Tamayo P, Mootha VK et al. Gene set enrichment analysis: a knowledge-based approach for interpreting genome-wide expression profiles, Proc Natl Acad Sci U S A 2005;102:15545-15550.

3. Liberzon A, Birger C, Thorvaldsdottir H et al. The Molecular Signatures Database (MSigDB) hallmark gene set collection, Cell Syst 2015;1:417-425.
